# A Novel Dynamic Model Describing the Spread of the MERS-CoV and the Expression of Dipeptidyl Peptidase 4

**DOI:** 10.1155/2017/5285810

**Published:** 2017-08-15

**Authors:** Siming Tang, Wanbiao Ma, Peifan Bai

**Affiliations:** Department of Applied Mathematics, School of Mathematics and Physics, University of Science and Technology Beijing, Beijing 100083, China

## Abstract

The Middle East respiratory syndrome (MERS) coronavirus, a newly identified pathogen, causes severe pneumonia in humans. MERS is caused by a coronavirus known as MERS-CoV, which attacks the respiratory system. The recently defined receptor for MERS-CoV, dipeptidyl peptidase 4 (DPP4), is generally expressed in endothelial and epithelial cells and has been shown to be present on cultured human nonciliated bronchiolar epithelium cells. In this paper, a class of novel four-dimensional dynamic model describing the infection of MERS-CoV is given, and then global stability of the equilibria of the model is discussed. Our results show that the spread of MERS-CoV can also be controlled by decreasing the expression rate of DPP4.

## 1. Introduction

The Middle East respiratory syndrome (MERS) coronavirus, a newly identified pathogen, causes severe pneumonia in humans, with a mortality of nearly 44%. Human-to-human spread has been demonstrated, raising the possibility that the infection could become pandemic [1]. A colorized electron micrograph shows the coronavirus MERS-CoV acute viral respiratory illness that is characterized primarily by cough, fever, and shortness of breath and is sometimes associated with severe and potentially fatal complications such as pneumonia and kidney failure. The illness was first observed in June 2012 in Jiddah, Saudi Arabia, and soon afterward it was reported in other countries in the Middle East, including Jordan, Qatar, and the United Arab Emirates (UAE). It later was detected in Europe, including cases in France, Germany, Italy, and the United Kingdom; in the North African country of Tunisia; and in countries more distant from the Middle East, including China, Malaysia, South Korea, and the United States. The largest MERS outbreak outside Saudi Arabia occurred in 2015, when an individual who had recently traveled to the Middle East subsequently fell ill in South Korea, transmitting the disease to close contacts. The dissemination of the disease by infected travelers leaving the Middle East suggested that MERS had the potential to escalate into an international public health emergency. The possibility of a pandemic was thought to be impeded, however, by the limited ability of the disease to be passed from one person to another. MERS is caused by a coronavirus known as MERS-CoV, which attacks the respiratory system. The recently defined receptor for MERS-CoV, dipeptidyl peptidase 4 (DPP4), is generally expressed in endothelial and epithelial cells and has been shown to be present on cultured human nonciliated bronchiolar epithelium cells, providing further information on the respiratory tropism of MERS-CoV [2]. Symptoms of illness appear anytime from 2 to 14 days following infection. Cough, fever, and shortness of breath are the primary symptoms, but others such as diarrhea, nausea, vomiting, and myalgia (muscle pain) can also occur. In some persons, infection produces no symptoms or only mild cold-like symptoms, whereas in others, particularly in persons with underlying medical conditions, infection can produce severe illness [3].

It is well-known that dynamic models are still playing important roles in describing the interactions among uninfected cells, free viruses, and immune responses (see, e.g., [4–7]). A three-dimensional dynamic model for viral infection is proposed by Nowak et al. (see, e.g., [5–7]).

(1) T˙=λ−βvtTt−dTt,I˙=βvtTt−d1It,v˙=d1NIt−cvt.

In model (1), *T*(*t*), *I*(*t*), and *v*(*t*) denote the concentration of uninfected cells, infected cells, and free viruses at time *t*, respectively. The constant *λ* > 0 is the rate at which new uninfected cells are generated (from a pool of precursor cells). The constants *d* > 0 and *β* ≥ 0 are the death rate of uninfected cells and the rate constant characterizing infection of the cells, respectively. The constant *d*_1_ > 0 is the death rate of the infected cells due to either viruses or immune responses. The infected cells produce new viruses at the rate *d*_1_*N* during their life, on average having the length 1/*d*_1_, where *N* > 0 is some integer number. The constant *c* > 0 is the rate at which the viruses are cleared, and the average lifetime of a free virus is 1/*c*.

**Figure 1:**
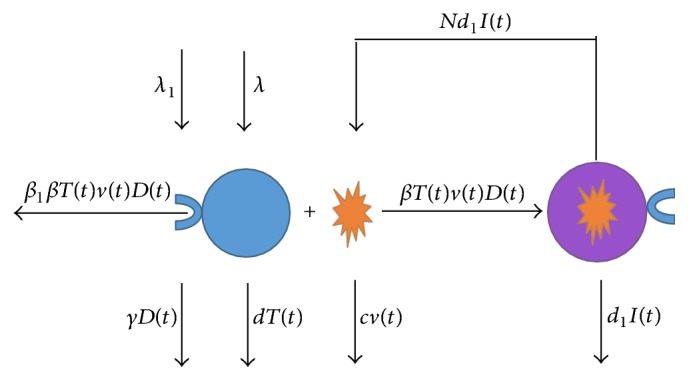
The interaction procedure between uninfected cells and MERS-CoV mediated by DPP4 receptors.

Figure 1 shows a interaction procedure between uninfected cells and MERS-CoV mediated by DPP4 receptors. Based on basic dynamic model (1) and Figure 1, we propose the following novel four-dimensional dynamic model which describes the spread of the MERS-CoV and the expression of DPP4:

(2) T˙=λ−βDtvtTt−dTt,I˙=βDtvtTt−d1It,v˙=d1NIt−cvt,D˙=λ1−β1βDtvtTt−γDt.

In model (2), *D*(*t*) represents the concentration of DPP4 on the surface of uninfected cells, which can be recognized by surface spike (S) protein of MERS-CoV (see, e.g., [8]). Infected cells are produced from uninfected cells and free viruses at the rate (*βD*(*t*))*v*(*t*)*T*(*t*). It is assumed that DPP4 is produced from the surface of uninfected cells at the constant rate *λ*_1_ > 0. DPP4 is destroyed, when free viruses try to infect uninfected cells, at the rate *β*_1_(*βD*(*t*))*v*(*t*)*T*(*t*), and is hydrolyzed at the rate *γD*(*t*). Here, *β*_1_ ≥ 0 and *γ* > 0 are constants. It is assumed that there is no undestroyed DPP4 on the surface of infected cells. All other parameters in model (2) have similar biological meanings to that in model (1).

The initial condition of model (2) is given as *T*(0) ≥ 0, *I*(0) ≥ 0, *v*(0) ≥ 0, and *D*(0) ≥ 0. It is not difficult to show that the solution (*T*(*t*), *I*(*t*), *v*(*t*), *D*(*t*)) with the initial condition is existent, unique, bounded, and nonnegative for all *t* ≥ 0 (in fact, it also has *T*(*t*) > 0 and *D*(*t*) > 0 for all *t* > 0). If *T*(0) > 0, *I*(0) > 0, *v*(0) > 0, and *D*(0) > 0, it is easily proven that the corresponding solution (*T*(*t*), *I*(*t*), *v*(*t*), *D*(*t*)) is positive for all *t* ≥ 0.

Furthermore, it can be easily shown that the set

(3) Ω=T,I,v,D ∣ 0≤T≤T0,  I≥0,  v≥0,  0≤D≤D0,  T+I+aNv≤λμ

is attractive and positively invariant with respect to model (2), where 0 < *a* < 1, *μ* = min⁡{*d*, (1 − *a*)*d*_1_, *c*}.

The purpose of the paper is to study local and global stability of the equilibria of model (2) by using Roth-Hurwitz criterion and constructing suitable Lyapunov function (see, e.g., [9–13]).

## 2. Local and Global Stability of the Equilibria

The basic reproductive ratio of the virus for model (2) is *R*_0_ = *Nβλλ*_1_/*cdγ*. Model (2) always has an infection-free equilibrium *E*_0_ = (*T*_0_, 0,0, *D*_0_) = (*λ*/*d*, 0,0, *λ*_1_/*γ*). If *R*_0_ > 1, model (2) also has unique infected equilibrium *E*^*∗*^ = (*T*^*∗*^, *I*^*∗*^, *v*^*∗*^, *D*^*∗*^), where, for *β*_1_ = 0, *v* = *v*^*∗*^ = *dγ*(*R*_0_ − 1)/*βλ*_1_, for *β*_1_ > 0, *v* = *v*^*∗*^ > 0 is the positive root of the equation *ββ*_1_*c*^2^*v*^2^ − *Ncβ*(*λ*_1_ + *λβ*_1_)*v* + *Ncdγ*(*R*_0_ − 1) = 0, and

(4) T∗=λd−cv∗Nd,I∗=cv∗Nd1,D∗=cNβT∗,v∗=Nβλ1+λβ1−N2β2λ1−λβ12+4Nβ1βcdγ2ββ1c.

First, we have the following result.

**Theorem 1 .**

With respect to the set *Ω*_1_ = {(*T*, *I*, *v*, *D*)∣(*T*, *I*, *v*, *D*) ∈ *Ω*, *T* > 0, *D* > 0}, the infection-free equilibrium *E*_0_ = (*T*_0_, 0,0, *D*_0_) is globally asymptotically stable when *R*_0_ < 1 and globally attractive when *R*_0_ = 1.

**Proof**

At any equilibrium (*T*, *I*, *v*, *D*), Jacobian matrix of model (2) is

(5) $$\begin{array}{c} J=\left(\begin{array}{cccc} -\beta vD-d & 0 & -\beta TD & -\beta vT\\ \beta vD & -d_{1} & \beta TD & \beta vT\\ 0 & Nd_{1} & -c & 0\\ -\beta_{1}\beta vD & 0 & -\beta_{1}\beta TD & -\beta_{1}\beta vT-\gamma \end{array}\right). \end{array}$$

By simple computations, we can get that the characteristic equation at *E*_0_ is *f*(*ρ*) = (*ρ* + *d*)(*ρ* + *γ*)[*ρ*^2^ + (*c* + *d*_1_)*ρ* + *cd*_1_(1 − *R*_0_)] = 0. Clearly, if *R*_0_ < 1, all roots of *f*(*ρ*) = 0 have negative real parts. Hence, *E*_0_ is local asymptotic stability by Routh-Hurwitz criterion. If *R*_0_ = 1, *f*(*ρ*) = 0 has the zero root *ρ* = 0 and three negative roots. Hence, *E*_0_ is linearly stable.

Construct the Lyapunov function as follows:

(6) UT,I,v,D=T0TT0−1−ln⁡TT0+1+β1I+1+β1Nv+D0DD0−1−ln⁡DD0.

It is clear that *U* is continuous on *Ω*_1_ and positive definite with respect to *E*_0_ and satisfies condition (ii) of Definition 1.1 in [14] or Lemma 3.1 in [15] on ∂*Ω* = *Ω*∖*Ω*_1_. Calculating the derivative of *U* along the solutions of model (2), we have, for *t* ≥ 0,

(7) dUdtλ−βDvT−dT−T0λT−βDv−d+βDvT+β1βDvT−1+β1d1I+1+β1Nd1NI−cvN−β1cvN+λ1−β1βDvT−γD−D0λ1D−β1βvT−γ=λ+dλd−dT−λT0T+λ1+γλ1γ−γD−λ1D0D+βDvT0−cvN+β1βD0vT−β1cvN=λ2−TT0−T0T+λ12−DD0−D0D+vβDT0−cN+β1vβD0T−cN≤λ2−TT0−T0T+λ12−DD0−D0D+cNR0−11+β1v.

Clearly, *dU*/*dt* ≤ 0 on *Ω*_1_ by *R*_0_ ≤ 1. Define *Q* = {*dU*/*dt* = 0∣(*T*, *I*, *v*, *D*) ∈ *Ω*, *U*(*T*, *I*, *v*, *D*) < +*∞*}. Let *M* be the largest subset in *Q* which is invariant with respect to the set model (2). Hence, we have that *M* ⊂ *Q* ⊂ {(*T*, *I*, *v*, *D*)∣(*T*, *I*, *v*, *D*) ∈ *Ω*, *T* = *T*_0_, *D* = *D*_0_}. From the invariance of *M* and model (2), we can easily show that *M* = {*E*_0_}. Therefore, it follows from Theorem 1.2 in [14] or Lemma 3.1 in [15] that *E*_0_ is globally attractive. This completes the proof.

For local and global stability of the infected equilibrium *E*^*∗*^, we have the following result.

**Theorem 2 .**

With respect to the set *Ω*_2_ = {(*T*, *I*, *v*, *D*)∣(*T*, *I*, *v*, *D*) ∈ *Ω*, *T* > 0, *I* > 0, *v* > 0, *D* > 0}, the infected equilibrium *E*^*∗*^ is locally asymptotically stable when *R*_0_ > 1. In addition, if (2*dγμa*)^2^ ≥ *β*_1_*β*^2^*λ*_1_*λ*^3^*N*^2^, where 0 < *a* < 1, *μ* = min⁡{*d*, (1 − *a*)*d*_1_, *c*}, the infected equilibrium *E*^*∗*^ is globally asymptotically stable.

**Proof**

The characteristic equation at of model (2) at *E*^*∗*^ is

(8) $$\begin{array}{c} g\left(\;\rho \;\right)=\rho^{4}+a_{3}\rho^{3}+a_{2}\rho^{2}+a_{1}\rho +a_{0}=0, \end{array}$$

where

(9) a0=T∗βcdv∗β1d1+D∗βcγv∗d1,a1=T∗βcv∗β1d1+T∗βdv∗β1d1+D∗βcγv∗+D∗βcv∗d1+D∗βγv∗d1+βcdv∗β1+cdγ+dγd1,a2=T∗βcv∗β1+T∗βdv∗β1+T∗βv∗β1d1+D∗βcv∗+D∗βγv∗+D∗βv∗d1+cd+cγ+dγ+dd1+γd1,a3=β1βv∗T∗+βv∗D∗+c+d+γ+d1.

It is obvious that *a*_*i*_ > 0  (*i* = 0,1, 2,3). Furthermore, by using Matlab program, it can been shown that Δ_3_ = *a*_1_*a*_2_*a*_3_ − *a*_1_^2^ − *a*_3_^2^*a*_0_ has 200 items in which all items are positive. Hence, *E*^*∗*^ is local asymptotic stability by Routh-Hurwitz criterion.

Construct the Lyapunov function as follows:

(10) W=β1T∗TT∗−1−ln⁡TT∗+β1I∗II∗−1−ln⁡II∗+β1Nv∗vv∗−1−ln⁡vv∗+D∗DD∗−1−ln⁡DD∗.

It is clear that *W* is continuous on *Ω*_2_ and positive definite with respect to *E*^*∗*^ and satisfies condition (ii) of Definition 1.1 in [14] or Lemma 3.1 in [15] on ∂*Ω* = *Ω*∖*Ω*_2_. Calculating the derivative of *W* along the solutions of model (2), we have, for *t* ≥ 0,

(11) dWdt=β1λ−dT−β1T∗λT−βDv−d−β1I∗βDvTI−d1−β1v∗d1Iv−cN+λ1−β1βDvT−γD−D∗λ1D−β1βTv−γ−β1cvN=β1βD∗v∗T∗+2β1dT∗−β1dT−β1T∗TβD∗v∗T∗+dT∗+β1βDvT∗−β1βDvTI∗I+β1d1I∗−β1d1Iv∗v+β1cv∗N+β1βD∗v∗T∗+2γD∗−β1βDvT−γD−D∗Dβ1βD∗v∗T∗+γD∗+β1βD∗Tv−β1cvN=2β1dT∗−β1dT−β1T∗TdT∗+2γD∗−γD−D∗DγD∗+2β1βD∗v∗T∗+β1cv∗N+β1d1I∗−β1βD∗v∗T∗D∗D−β1βD∗v∗T∗T∗T−β1βDvTI∗I−β1d1Iv∗v+β1βDvT∗+β1βD∗Tv−β1cvN−β1βDvT=β1dT∗2−TT∗−T∗T+γD∗2−DD∗−D∗D+β1d1I∗4−T∗T−D∗D−βTvDd1I−Iv∗I∗v+β1βvTD∗+T∗D−TD−cβN=β1dT∗2−TT∗−T∗T+γD∗2−DD∗−D∗D+β1d1I∗4−T∗T−D∗D−TvDI∗T∗v∗D∗I−Iv∗I∗v+β1βvTD∗+T∗D−TD−T∗D∗=−β1dTT−T∗2−γDD−D∗2−ββ1vD−D∗T−T∗+β1d1I∗4−T∗T−D∗D−TvDI∗T∗v∗D∗I−Iv∗I∗v.

Since the arithmetical mean is greater than or equal to the geometrical mean, we have that *T*^*∗*^/*T* + *D*^*∗*^/*D* + *TvDI*^*∗*^/*T*^*∗*^*v*^*∗*^*D*^*∗*^*I* + *Iv*^*∗*^/*I*^*∗*^*v* − 4 ≤ 0, for any *T*, *I*, *v*, *D* > 0, and that *T*^*∗*^/*T* + *D*^*∗*^/*D* + *TvDI*^*∗*^/*T*^*∗*^*v*^*∗*^*D*^*∗*^*I* + *Iv*^*∗*^/*I*^*∗*^*v* − 4 = 0 only if *T*^*∗*^/*T* = *D*^*∗*^/*D* = *TvDI*^*∗*^/*T*^*∗*^*v*^*∗*^*D*^*∗*^*I* = *Iv*^*∗*^/*I*^*∗*^*v*. Thus, we have *T* = *T*^*∗*^, *D* = *D*^*∗*^.

On the other hand, notice the inequality in [16]:

(12) $$\begin{array}{c} -xz^{2}+yz\leq -\frac{1}{2}xz^{2}+\frac{y^{2}}{2x}\;\left(x>0,\;\;y\geq 0,\;\;z\geq 0\right). \end{array}$$

We have

(13) −β1dTT−T∗2−12ββ1vD−D∗T−T∗−γDD−D∗2−12ββ1vD−D∗T−T∗≤−β1d2TT−T∗2+Dββ1v28γT−T∗2−γ2DD−D∗2+Tββ1v28β1dD−D∗2=−β1d2T−Dββ1v28γT−T∗2−γ2D−Tββ1v28β1dD−D∗2.

Note that the inequalities *β*_1_*d*/2*T* − *D*(*ββ*_1_*v*)^2^/8*γ* ≥ 0 and *γ*/2*D* − *T*(*ββ*_1_*v*)^2^/8*β*_1_*d* ≥ 0 are equivalent to the inequality 4*dγ* ≥ *β*_1_*β*^2^*TDv*^2^. Since *T*(*t*) ≤ *T*_0_, *D* ≤ *D*_0_, and *v*(*t*) ≤ *λN*/*μa* for all *t* ≥ 0, we have that the inequality 4*dγ* ≥ *β*_1_*β*^2^*TDv*^2^ holds, if the condition (2*dγμa*)^2^ ≥ *β*_1_*β*^2^*λ*_1_*λ*^3^*N*^2^ in Theorem 2 is satisfied. Therefore, *dW*/*dt* ≤ 0 on *Ω*_2_.

Define *Q* = {*dW*/*dt* = 0∣(*T*, *I*, *v*, *D*) ∈ *Ω*, *W*(*T*, *I*, *v*, *D*) < +*∞*}. Let *M* be the largest subset in *Q* which is invariant with respect to the set of model (2). Hence, we have that *M* ⊂ *Q* ⊂ {(*T*, *I*, *v*, *D*)∣(*T*, *I*, *v*, *D*) ∈ *Ω*, *T* = *T*^*∗*^, *D* = *D*^*∗*^}. From the invariance of *M* and model (2), we can also show that *M* = {*E*^*∗*^}. Hence, it follows from Theorem 1.2 in [14] or Lemma 3.1 in [15] that *E*^*∗*^ is globally attractive. This completes the proof.

## 3. Simulations and Conclusions

**Figure 2:**
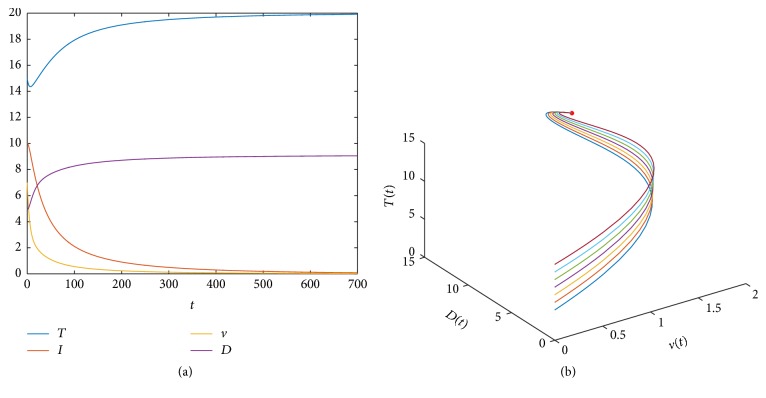
(a) The trajectory of model (2) when *E*_0_ = (20,0, 0,9.0909) and *R*_0_ = 0.90909 < 1. (b) The orbits of model (2) when *E*^*∗*^ = (14.142,5.8579,1.4645,14.142) and *R*_0_ = 2 > 1.

Let us first give some numerical simulations on the orbits of model (2). Take the following a set of parameters, *λ* = *λ*_1_ = 1, *β* = 0.001, *d* = *d*_1_ = 0.05, *N* = 1, *c* = 0.2, *β*_1_ = 1, and *γ* = 0.11. We can compute the values of the infection-free equilibrium *E*_0_ and the basic reproductive ratio, *E*_0_ = (20,0, 0,9.0909) and *R*_0_ = 0.90909 < 1.  Figure 2(a) shows the trajectory of model (2) with suitable initial condition, which shows that the infection-free equilibrium *E*_0_ is asymptotically stable.

Let us take *γ* = 0.05, and all the other parameters are the same as above. We can also compute the values of the infection-free equilibrium *E*_0_, the infected equilibrium *E*^*∗*^, and the basic reproductive ratio, *E*_0_ = (20,0, 0,20), *E*^*∗*^ = (14.142,5.8579,1.4645,14.142), and *R*_0_ = 2 > 1.  Figure 2(b) shows orbits of model (2) with suitable initial conditions, which shows that the infected equilibrium *E*^*∗*^ is asymptotically stable. We would like to point out here that, based on the numerical simulations, the condition (2*dγμa*)^2^ ≥ *β*_1_*β*^2^*λ*_1_*λ*^3^*N*^2^ may be further weakened or even removed.

Finally, by using the basic reproductive ratio *R*_0_ = *Nβλλ*_1_/*cdγ*, let us give some simple discussions on the interactions between the protein DPP4 and the virus infection. Usually, in the absence of any drug treatment, all the parameters in model (2) and the corresponding basic reproductive ratio *R*_0_ can be regarded as relatively fixed constants. If some drug treatment measures are taken, the effectiveness of the treatment can be reflected in the regulation of the parameter *γ*. For example, by increasing the value of *γ*, the value of the basic reproductive ratio of *R*_0_ can be changed from greater than 1 to less than 1. In the numerical simulations in this section, Figure 2(b) shows that the virus infection will be persistent, when *γ* = 0.05 and *R*_0_ = 2 > 1. If increasing *γ* from *γ* = 0.05 to *γ* = 0.11, Figure 2(a) shows that the virus infection can be controlled, since *R*_0_ = 0.9090 < 1.

## References

[1] Zhao J., Li K., Wohlford-Lenane C. (2014). Rapid generation of a mouse model for Middle East respiratory syndrome. *Proceedings of the National Academy of Sciences of the United States of America*.

[2] De Wit E., Rasmussen A. L., Falzarano D. (2013). Middle East respiratory syndrome coronavirus (MERSCoV) causes transient lower respiratory tract infection in rhesus macaques. *Proceedings of the National Academy of Sciences of the United States of America*.

[3] Rogers K. MERSE. http://academic.eb.com/EBchecked/topic//.

[4] Anderson R. M., May R. M. (1991). *Infectious Diseases of Humans: Dynamics and Control*.

[5] Nowak M. A., Bangham C. R. M. (1996). Population dynamics of immune responses to persistent viruses. *Science*.

[6] Nowak M. A., May R. M. (2000). *Virus Dynamics: Mathematics Principles of Immunology and Virology*.

[7] Perelson A. S., Nelson P. W. (1999). Mathematical analysis of HIV-1 dynamics in vivo. *SIAM Review*.

[8] Mille J. K., Whittaker G. R. (2014). Host cell entry of Middle East respiratory syndrome coronavirus after two-step, furin-mediated activation of the spike protein. *Proceedings of the National Academy of Sciences of the United States of America*.

[9] Korobeinikov A. (2004). Global properties of basic virus dynamics models. *Bulletin of Mathematical Biology*.

[10] Korobeinikov A. (2009). Global asymptotic properties of virus dynamics models with dose-dependent parasite reproduction and virulence and non-linear incidence rate. *Mathematical Medicine and Biology*.

[11] McCluskey C. C. (2010). Complete global stability for an SIR epidemic model with delay-distributed or discrete. *Nonlinear Analysis. Real World Applications*.

[12] Huang G., Ma W., Takeuchi Y. (2009). Global properties for virus dynamics model with Beddington-DeAngelis functional response. *Applied Mathematics Letters. An International Journal of Rapid Publication*.

[13] Li F., Ma W., Jiang Z., Li D. (2015). Stability and Hopf bifurcation in a delayed HIV infection model with general incidence rate and immune impairment. *Computational and Mathematical Methods in Medicine*.

[14] Wolkowicz G. S., Lu Z. Q. (1992). Global dynamics of a mathematical model of competition in the chemostat: general response functions and differential death rates. *SIAM Journal on Applied Mathematics*.

[15] Saito Y., Hara T., Ma W. (1999). Necessary and sufficient conditions for permanence and global stability of a Lotka-Volterra system with two delays. *Journal of Mathematical Analysis and Applications*.

[16] Qin Y. X., Wang M. Q., Wang L. (1981). *Theory of Motion Stability and Their Applications*.

